# Potential of Unenhanced Ultra-Low-Dose Abdominal Photon-Counting CT with Tin Filtration: A Cadaveric Study

**DOI:** 10.3390/diagnostics13040603

**Published:** 2023-02-07

**Authors:** Henner Huflage, Jan-Peter Grunz, Theresa Sophie Patzer, Pauline Pannenbecker, Philipp Feldle, Stephanie Tina Sauer, Bernhard Petritsch, Süleyman Ergün, Thorsten Alexander Bley, Andreas Steven Kunz

**Affiliations:** 1 Department of Diagnostic and Interventional Radiology, University Hospital Würzburg, Oberdürrbacher Straße 6, 97080 Würzburg, Germany; 2 Institute of Anatomy and Cell Biology, University of Würzburg, Koellikerstraße 6, 97070 Würzburg, Germany

**Keywords:** spectral shaping, tin prefiltration, abdominal imaging, ultra-low-dose CT, urinary calculi, photon-counting

## Abstract

Objectives: This study investigated the feasibility and image quality of ultra-low-dose unenhanced abdominal CT using photon-counting detector technology and tin prefiltration. Materials and Methods: Employing a first-generation photon-counting CT scanner, eight cadaveric specimens were examined both with tin prefiltration (Sn 100 kVp) and polychromatic (120 kVp) scan protocols matched for radiation dose at three different levels: standard-dose (3 mGy), low-dose (1 mGy) and ultra-low-dose (0.5 mGy). Image quality was evaluated quantitatively by means of contrast-to-noise-ratios (CNR) with regions of interest placed in the renal cortex and subcutaneous fat. Additionally, three independent radiologists performed subjective evaluation of image quality. The intraclass correlation coefficient was calculated as a measure of interrater reliability. Results: Irrespective of scan mode, CNR in the renal cortex decreased with lower radiation dose. Despite similar mean energy of the applied x-ray spectrum, CNR was superior for Sn 100 kVp over 120 kVp at standard-dose (17.75 ± 3.51 vs. 14.13 ± 4.02), low-dose (13.99 ± 2.6 vs. 10.68 ± 2.17) and ultra-low-dose levels (8.88 ± 2.01 vs. 11.06 ± 1.74) (all *p* ≤ 0.05). Subjective image quality was highest for both standard-dose protocols (score 5; interquartile range 5–5). While no difference was ascertained between Sn 100 kVp and 120 kVp examinations at standard and low-dose levels, the subjective image quality of tin-filtered scans was superior to 120 kVp with ultra-low radiation dose (*p* < 0.05). An intraclass correlation coefficient of 0.844 (95% confidence interval 0.763–0.906; *p* < 0.001) indicated good interrater reliability. Conclusions: Photon-counting detector CT permits excellent image quality in unenhanced abdominal CT with very low radiation dose. Employment of tin prefiltration at 100 kVp instead of polychromatic imaging at 120 kVp increases the image quality even further in the ultra-low-dose range of 0.5 mGy.

## 1. Introduction

In recent years, spectral shaping via tin prefiltration has gained increasing recognition in CT research, albeit primarily focused on the paranasal sinus, lung and skeletal system [1,2,3,4,5,6,7]. With regard to abdominal imaging, the use of tin filters is still less common, although studies employing energy-integrating-detector CT systems have previously shown substantial effects here, too [7,8,9,10]. Latest investigations with first-generation photon-counting detector CT (PCD-CT) systems suggest potential for further dose reduction in various imaging tasks [11,12,13,14,15]. However, the actual impact of tin prefiltration in ultra-low-dose photon-counting abdominal CT scans is still to be determined. A typical clinical application for unenhanced low-dose CT of the abdomen is for detection of urinary calculi [16,17,18]. In the case of urolithiasis, CT is considered superior to other imaging techniques such as ultrasound with sensitivity reported at 97% and specificity at 95% [19,20,21]. The prevalence of symptomatic kidney stones in women aged 20–39 reached 6.1% in 2013 and even in childhood increasing incidences are nowadays observed [22,23]. With recurrence rates of approximately 50% and peak prevalence in working age adults, nephrolithiasis carries a significant socioeconomic burden [24]. That being said, effects of repetitive radiation exposure constitute a relevant concern. While being disputed, individual estimations associate up to 14,500 cancer deaths per year to CT examinations [25,26]. In this regard, ACR guidelines explicitly demand CT scans to exploit dose reduction techniques in order to induce a radiation burden of no more than 3 mSv [18]. Recently introduced PCD-CT bears the principal capacity of combining CT-imaging with spectral analysis for all scans performed. Employing spectral shaping with tin filtration has been reported to significantly reduce radiation dose for low-dose scans at the cost of currently limited spectral functionality with regards to renal stone characterization, among others [27,28].

For this study, we hypothesized that tin prefiltration at 100 kVp permits superior objective and subjective image quality in ultra-low-dose abdominal PCD-CT than standard polychromatic examinations with 120 kVp.

## 2. Methods

Eight cadaveric specimens fixed in formalin were obtained from our university’s institute of anatomy after permission for this experimental study was granted by the local ethics committee. No further written informed consent was required, since agreement had been given prior to death that these cadaveric specimens were to be subjected to scientific investigations. The study was planned and executed with respect to organizational statutes, as well as federal legislation.

### 2.1. Scan and Image Reconstruction Parameters

All images were acquired with a commercial first-generation PCD-CT system (Naeotom Alpha, Siemens Healthcare GmbH, Forchheim, Germany; software version VA40). As per clinical standard, cadaveric bodies were examined with arms elevated above the head in supine position. Scan ranges were planned based on anterior-posterior topograms with identical scan lengths for all six examination protocols applied to each specimen. Each cadaveric specimens was scanned with three pairs of tin prefiltration and polychromatic scan protocols, which were matched for radiation dose. Target volume CT dose indices (CTDI_vol_) for the respective pairs were 3 mGy (referred to as standard-dose), 1 mGy (low-dose) and 0.5 mGy (ultra-low-dose). All examinations were performed in macro mode with a collimation of 144 × 0.4 mm, resulting in a total collimation of 57.6 mm. Tube potential was 100 kVp for tin-filtered examinations and 120 kVp for polychromatic scans. Helical pitch factor was set to 1.0 and selected gantry rotation time was 0.5 s for all studies. Examinations were performed with active automatic dose modulation, aiming to resemble a realistic clinical scenario. Acquisition parameters are summarized in Table 1.

**Figure 1:**
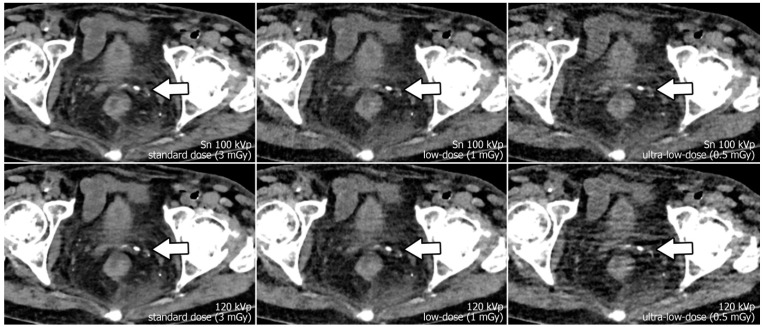
Axial view of the left distal ureter (arrow). Note the impaired image quality due to hypodense streak artifacts in polychromatic scans at 120 kVp (lower row), especially with ultra-low radiation dose. (The arrow marks the left ureter with an adjacent phlebolite.)

**Figure 2:**
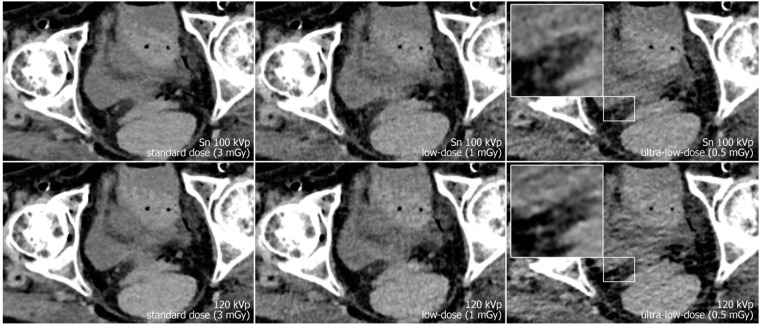
Axial view of the right distal ureter (magnification) in a different specimen. Again, the image quality is reduced and demarcability of the ureter is impaired, especially in ultra-low-dose polychromatic imaging compared to the dose-matched protocol with tin filtration.

Reconstruction of datasets followed identical parameters: slice thickness and increment were set to 3 mm each, and the field of view to 400 mm with a 512 × 512 pixel matrix. A soft tissue convolution kernel (Br36) was applied with the highest strength level of a dedicated quantum iterative reconstruction algorithm (QIR 4). For polychromatic scans, “T3D” mode was enabled, which includes photon energies between 20 and 120 keV [29]. Window settings were pre-set to 300/40 Hounsfield units (width/center) were employed for dedicated analysis of soft tissue. However, readers were given the possibility to adjust these settings at will during image quality assessment. Examples regarding differences in image quality of the pelvic region between acquisition protocols are provided in Figure 1, while Figure 2 illustrates the encumbered demarcability of the lower Ureter in ultra-low-dose scans.

### 2.2. Objective Image Analysis

A radiologist with six years of experience positioned standardized regions of interest bilaterally in the renal cortex and in subcutaneous fat on axial reconstructions. For each region of interest, mean density in Hounsfield units and associated standard deviation were documented. Contrast-to-noise ratios (CNR) were calculated according to Yel et al. for both kidneys [30]:

$$\mathrm{CNR}=\frac{\mathrm{mean}\;\mathrm{attenuation}\;\left(\mathrm{renal}\;\mathrm{cortex}\right)-\;\mathrm{mean}\;\mathrm{attenuation}\;\left(\mathrm{fat}\right)}{\mathrm{standard}\;\mathrm{deviation}\;\left(\mathrm{fat}\right)}$$

### 2.3. Subjective Image Analysis

Three radiologists with at least five years of experience in abdominal CT imaging analyzed all datasets independently using dedicated picture archiving and communication software (Merlin, Phönix-PACS, Freiburg im Breisgau, Germany) and monitors certified for diagnostic viewing (30-inch diameter, RadiForce RX660, EIZO, Hakusan, Japan). Acquisition and reconstruction parameters were not revealed to readers who were tasked with evaluating overall image quality on an equidistant five-point scale with a rating of 5 indicating “excellent image quality; no impairment of image quality for diagnostic purposes due to image noise” and 1 indicating “inadequate image quality; image quality insufficient for diagnostic purposes due to substantial image noise”.

### 2.4. Statistics

Statistical analyses were performed with dedicated software (SPSS Statistics Version 28, IBM, Armonk, NY, USA) and *p*-values of less than 0.05 were assumed to indicate significant differences. For normal distributions observed with the Kolmogorov-Smirnov test, the respective item is reported as mean ± standard deviation, while categorical variables are presented as absolute and relative frequencies with median values and interquartile ranges (IQR). Friedman’s rank-based ANOVA was utilized for comparison of ordinal variables and one-way repeated measures ANOVA was employed for comparing cardinal data. In both cases, pairwise post-hoc tests were performed with Bonferroni-correction for multiple comparisons. The intraclass correlation coefficient (ICC) was computed for assessment of inter-reader reliability within a 95% confidence interval. ICC values were specified following Koo and Li (excellent: 1.00–0.90; good: 0.90–0.75; moderate: 0.75–0.50; poor: <0.50) [31].

## 3. Results

### 3.1. Radiation Doses

**Figure 3:**
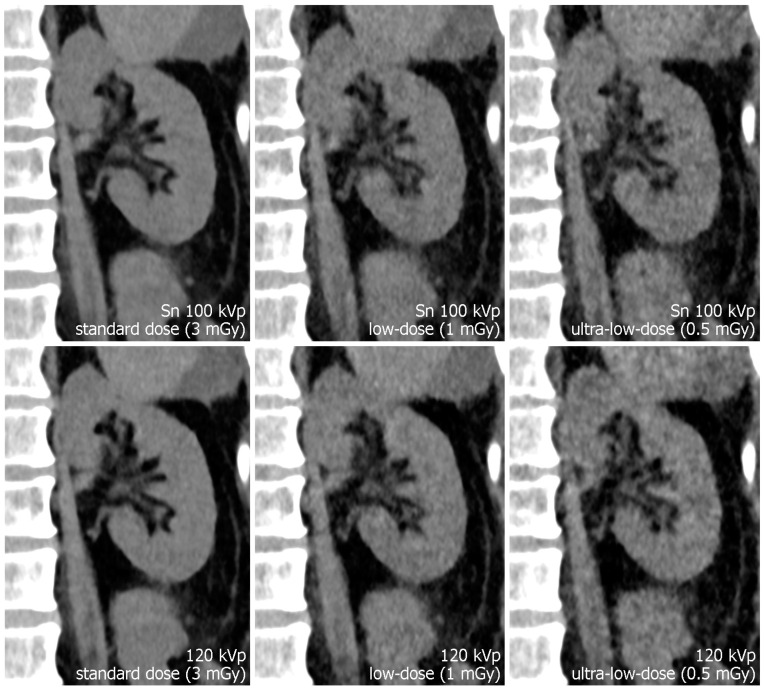
Para-coronal images displaying the left kidney. The renal pelvis is not dilated but pelvic structures remain assessable even in ultra-low-dose images. Note the increased noise in the 120 kVp ultra-low-dose study compared to the dose-matched protocol with tin filtration.

**Figure 4:**
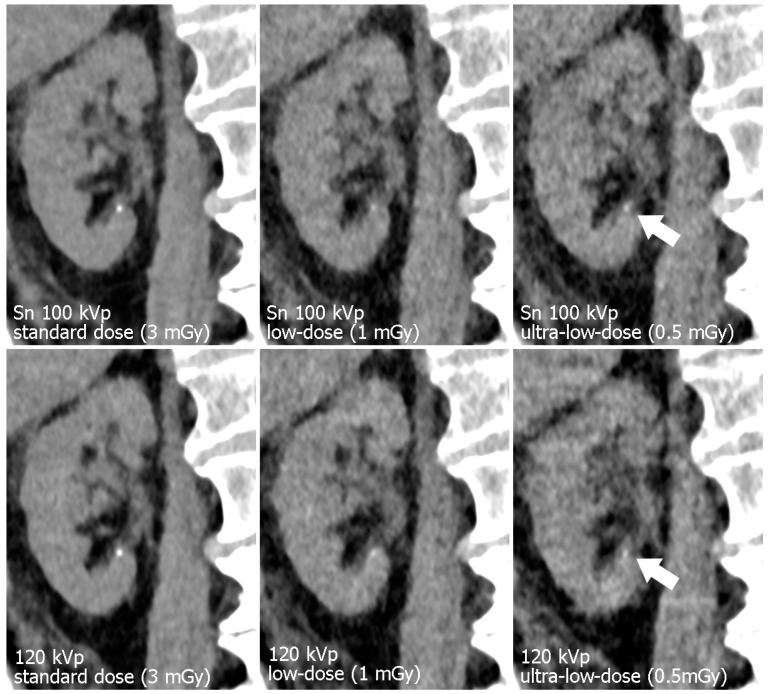
Para-coronal images of the right kidney display a minuscule calcification (arrow). Note the decrease in image quality with lower radiation dose. Organ margins and the calcification appear sharper with tin filtration ultra-low-dose imaging.

CTDI_vol_ showed minor variations between specimens, as is to be expected with activated automatic tube modulation and different constitutions of the cadaveric specimens. The standard-dose tin-filtered protocol was associated with a mean CTDI_vol_ of 3.38 ± 0.53 mGy, while the respective polychromatic scan protocol resulted in a mean CTDI_vol_ of 3.53 ± 0.62 mGy (*p* = 0.108). Referring to the low-dose level, mean CTDI_vol_ of Sn 100 kVp examinations was lower than of polychromatic 120 kVp scans (1.12 ± 0.18 vs. 1.21 ± 0.21 mGy; *p* = 0.007). For ultra-low-dose studies, tin prefiltration resulted in a mean CTDI_vol_ of 0.55 ± 0.09 mGy, whereas polychromatic imaging produced a matching radiation dose of 0.56 ± 0.09 mGy (*p* = 0.168). Table 2 provides a detailed display of individual dose parameters per specimen. Figure 3; Figure 4 illustrate the effects of dose reduction and spectral shaping regarding demarcability of kidney organ structures and detection of urinary calculi.

### 3.2. Objective Image Quality

Irrespective of tube voltage, CNR in the renal cortex decreased with lower radiation dose. For standard-dose examinations, mean CNR of studies with tin prefiltration was 17.75 ± 3.51 vs. 14.13 ± 4.02 in polychromatic 120 kVp imaging. For low-dose scans, CNR was measured at 13.99 ± 2.6 in Sn 100 kVp and 10.68 ± 2.17 for 120 kVp. With regards to ultra-low-dose scans, CNR was 11.06 ± 1.74 with tin prefiltration and 8.88 ± 2.01 in polychromatic scans. While CNR scores in the scan protocols with tin prefiltration were higher compared to their dose-equivalent polychromatic counterparts (all *p* < 0.001), no substantial difference was detected between Sn 100 kVp low-dose scans and 120 kVp standard-dose scans without tin filtration, as well as between ultra-low-dose studies with tin filter and polychromatic low-dose imaging (both *p* > 0.999).

### 3.3. Subjective Image Quality

Standard-dose scans received the highest ratings among all scan protocols, irrespective of tube potential. Subjective image quality ratings were comparable among both standard-dose (*p* = 0.903) and both low-dose protocols (*p* = 0.527). Contrarily, in the ultra-low-dose range, tin-filtered studies at 100 kVp were perceived to be superior to polychromatic 120 kVp scans (*p* < 0.005). A single-measures ICC indicated good interrater reliability with 0.844 (95% confidence interval 0.763–0.906; *p* < 0.001). Objective and subjective image quality parameters are presented in Table 3.

## 4. Discussion

This experimental study investigated the feasibility of non-contrast, ultra-low-dose photon-counting detector abdominal CT of the abdomen and evaluated whether tin filtration or polychromatic imaging is superior regarding image quality in this extreme setting. We were able to show that dose-reduced scans of the abdomen provide sufficient image quality for future patient scans and can thus issue a detailed protocol recommendation. Additionally, this study suggests that employing tin prefiltration in combination with the photon-counting detector is beneficiary especially in the case of ultra-low-dose imaging, where the tin filter increased the CNR to match the results achieved with higher radiation dose in the 120 kVp group. Notably, subjective image quality ratings indicated no substantial differences between tin-filtered and polychromatic scan protocols of the next higher dose level.

The acquired 120 kVp scans were reconstructed in a polychromatic image mode that accumulated all measured photons above the lowest energy threshold of 20 keV in order to warrant comparability to conventional imaging [32]. Contrarily, virtual monoenergetic reconstructions are not applicable after hardening the photon spectrum by tin prefiltration even though they, too, were reconstructed as polychromatic images (termed “T3D” by the vendor). The latter closely resembles a conventional reconstruction approach performed with commonly used energy-integrating detector systems [33]. Thus, improved image quality based on tin filtered protocols can be achieved if material decomposition is not the aim. However, with both techniques described, it is possible to stay well below the dedicated 3 mGy limit recommended by the ACR and thus maintain a reasonable radiation exposure in this typically young and thus vulnerable patient group.

In abdominal PCD-CT imaging, a recent study suggested that the standard virtual monoenergetic reconstruction setting of 60 keV was inferior to polychromatic 120 kVp imaging with regards to noise in all iterative reconstruction strength levels [32]. Furthermore, pre-clinical studies have suggested radical dose saving potential derived from the more efficient photon-counting detector technology [29,33]. First patient studies on non-contrast, low-dose scans of the abdomen with tin filtration showed superiority of the PCD-CT system over state-of-the-art scanners with energy-integrating detector detectors [34]. However, dedicated studies evaluating the specific influence of spectral shaping via tin prefiltration in PCD-CT are lacking.

Compared to the current generation of dual-source energy-integrating detector CT systems, which are equipped with a 0.6 mm tin filter, the investigated PCD-CT system is fitted with a 0.4 mm filter. In consequence, it remains unclear whether study results are unconditionally transferable under these conditions. In addition, features such as electronic denoising, virtual monoenergetic imaging and a new iterative reconstruction algorithm have been introduced with the PCD-CT system, hence necessitating further dedicated studies [32]. To the authors’ best knowledge, the present investigation is the first to directly compare ultra-low-dose scan protocols for the abdomen with and without tin prefiltration on a PCD-CT system.

Several limitations must be considered for this study: First, the cadaveric specimens examined were of normal constitution; none was morbidly obese or cachectic. Second, it must be presumed that image quality and ureter assessability were influenced by varying degrees of pneumatosis and intra-vasal air due to the formalin fixation process. Third, three cadaveric specimens had undergone previous hip replacement, impairing locoregional assessability of the distal ureters. Fourth, scans were associated with minor variances among cadaveric specimens regarding CTDI_vol_ due to automatic dose modulation. Lastly, the minimum tube current applicable in a 120 kVp setting is 5 mAs, hence restricting the achievable dose reduction compared to protocols employing spectral shaping.

## 5. Conclusions

Photon-counting detector CT allows for excellent image quality in unenhanced abdominal CT with very low radiation dose for detecting urinary calculi. Employment of tin prefiltration at 100 kVp instead of polychromatic imaging at 120 kVp increases subjective and objective image quality even further in the ultra-low-dose range of 0.5 mGy.

## Figures and Tables

**Table 1:** Summarized technical details of the acquired protocols. Apart from dose settings all parameters were maintained identical.

Protocol Parameters	Tin Prefiltration	Polychromatic Mode
Tube voltage	Sn 100 kVp	120 kVp
Targeted CTDI_vol_ [mGy]	3; 1; 0.5	
Tube current-time product [mAs]	Automatic	
Rotation time [s]	0.5	
Pitch factor	1.0	
Convolution kernel	Br36	
Iterative reconstruction strength	QIR Level 4	
Slice thickness [mm]	3	
Increment [mm]	3	

Note.–CTDI_vol_ = Volume CT dose index; Sn = with 0.4 mm tin filter.

**Table 2:** Volume CT dose indices with automatic dose modulation and vendor-specific image quality settings for each of the eight cadaveric specimens examined in this study. Notably, polychromatic imaging was associated with higher radiation dose than low-dose scans with tin prefiltration.

Mode	Tin Prefiltration (Sn 100 kVp)			Polychromatic Mode (120 kVp)		
Protocol	Standard Dose	Low Dose	Ultra-Low Dose	Standard Dose	Low Dose	Ultra-Low Dose
	CTDI_vol_ [mGy]			CTDI_vol_ [mGy]		
Specimen 1	2.73	0.9	0.44	2.75	0.94	0.44
Specimen 2	2.61	0.87	0.43	2.63	0.91	0.44
Specimen 3	3.22	1.07	0.53	3.35	1.13	0.53
Specimen 4	3.83	1.28	0.63	4.15	1.43	0.64
Specimen 5	3.00	0.98	0.49	3.07	1.07	0.5
Specimen 6	3.78	1.27	0.62	4.07	1.36	0.63
Specimen 7	3.78	1.23	0.62	3.97	1.36	0.62
Specimen 8	4.1	1.35	0.67	4.29	1.47	0.68
Mean CTDI_vol_ ± SD [mGy]	3.38 ± 0.53	1.12 ± 0.18	0.55 ± 0.09	3.53 ± 0.62	1.21 ± 0.21	0.56 ± 0.09

Note.–CTDI_vol_ = Volume CT dose index; SD = standard deviation; Sn = with 0.4 mm tin filter.

**Table 3:** Summary of objective and subjective image quality parameters.

Mode	Tin Prefiltration (Sn 100 kVp)			Polychromatic Mode (120 kVp)		
Protocol	Standard Dose	Low Dose	Ultra-Low Dose	Standard Dose	Low Dose	Ultra-Low Dose
Mean CNR ± SD	17.75 ± 3.51	13.99 ± 2.6	11.06 ± 1.74	14.13 ± 4.02	10.68 ± 2.17	8.88 ± 2.01
Median rating [IQR]	5 [5; 5]	4 [4; 4]	3 [3; 4]	5 [5; 5]	4 [4; 4]	3 [2; 3]

Note.–CNR = contrast-to-noise ratio; IQR = interquartile range; SD = standard deviation; Sn = with 0.4 mm tin filter.

## Data Availability

Data is made available upon reasonable request.

## Abbreviations and Acronyms

CNR	contrast-to-noise ratio
CTDI_vol_	volume computed tomography dose index
IQR	interquartile range
PCD-CT	photon-counting detector computed tomography
